# Graduates in Dentistry, 1880

**Published:** 1880-05

**Authors:** 


### Graduates In Dentistry 1880.

Below are given the names of the graduates of all the Dental Colleges in the United States for the year 1880, except Harvard Dental College, the commencement of which occurs in June:

### BALTIMORE COLLEGE OF DENTAL SURGERY.

The fortieth annual commencement of the Baltimore College of Dental Surgery was held March 4th, 1880.

The degree of D. D. S. was conferred on the following persons:

| NAME. | RESIDENCE. |
|---|---|
| C. James Barber | New York. |
| Frank A. Barrett | Dist. of Columbia. |
| John D. Basehore | Pennsylvania. |
| John S. Billopp | Maryland. |
| John H. Burnett | South Carolina. |
| Oscar Frederick Coe | New York. |
| Frederick H. Cole | New York. |
| Pastor A. Cooke | South America. |
| H. V. Desportes | South Carolina. |
| Josiah W. Foreman | Virginia. |
| James W. Gorden | Maryland. |
| Milton H. Gross | Pennsylvania. |
| William T. Harban | Maryland. |
| Joseph S. Hartman | Virginia. |
| William Hawkins | Tennessee. |
| Garnett L. Hills | Dist. of Columbia. |
| N. A. Hollinshead | Georgia. |
| B. Merrill R. Hopkinson | Maryland. |
| Louis C. F. Hugo | Dist. of Columbia. |
| Thomas May Hunter | North Carolina. |

| | |
|---|---|
| Albert B. King | Maryland. |
| Alexander Leeds | Maryland. |
| Frank P. Lewis | Pennsylvania. |
| Burrows Nelson | Dist. of Columbia. |
| Carl H. E. Obermuller | Germany. |
| John C. Oeland | South Carolina. |
| Elisha T. Payne | New York. |
| Samuel A. Peden | Pennsylvania. |
| James A. Pierce | Virginia. |
| Marion Pirkey | Virginia. |
| Joseph M. Quattlebaum | South Carolina. |
| Henry L. Rankin | Virginia. |
| Abraham V. Robbins | Pennsylvania. |
| J. Ryerson Smith | South Carolina. |
| Millard F. Thompson | Dist. of Columbia. |
| Louis A. Thurber | Louisiana. |
| John H. Twyman | Kentucky. |
| J. N. Van de Water | New York. |
| Louis G. Wietfeldt | Germany. |
| John L. Wolf, M. D | Dist. of Columbia. |

HONORARY DEGREE.

| | |
|---|---|
| Thomas Brian Gunning | New York. |

### OHIO COLLEGE OF DENTAL SURGERY.

The thirty-fourth annual commencement of the Ohio College of Dental Surgery was held March 3rd, 1880.

The degree of D. D. S. was conferred on the following persons:

| NAME. | RESIDENCE. |
|---|---|
| Ira Athern | Ohio. |
| Will B. Ames | Ohio. |
| David Arter | Ohio. |
| John O. Bockstoce | Pennsylvania. |
| F. O. Brake | Ohio. |
| J. M. Clyde | Kentucky. |
| G. M. Cole | Pennsylvania. |
| M. H. Fletcher | Indiana. |
| Hubert H. Gott | Ohio. |
| W. M. Garnett | Kentucky. |
| E. A. Galbrath | Ohio. |
| John McGarry | Ohio. |
| A. N. Hauck | Pennsylvania. |

| | |
|---|---|
| R. L. Hyde | Ohio. |
| A. W. Harlan | Illinois. |
| John H. Martindale | Minnesota. |
| H. L. Millikin | Ohio. |
| W. S. Myers | Indiana. |
| W. P. Merrill | Indiana. |
| W. C. Nesbitt | Kentucky. |
| S. W. Poland | Ohio. |
| Miss Annie Riley | Ohio. |
| A. J. Richter | Wisconsin. |
| C. M. Richey | Indiana. |
| E. H. Steckman | Missouri. |
| C. H. Snyder | Ohio. |
| S. S. Street | Ohio. |
| G. W. Sparrock | West Indies. |
| C. T. Wiant | Ohio. |
| B. W. Wikoff | Ohio. |
| Samuel Young | Tennessee. |

### PENNSYLVANIA COLLEGE OF DENTAL SURGERY.

The twenty-fourth annual commencement of the Pennsylvania College of Dental Surgery was held February 27th, 1880.

The degree of D. D. S. was conferred on the following graduates viz. :

| NAME. | RESIDENCE. |
|---|---|
| D. D. Atkinson, | Georgia. |
| Lewis S. Ayers, | New Jersey. |
| M. Allman, | Pennsylvania. |
| William C. Barrett, | New York. |
| F. W. Bennett, | Illinois. |
| Adolphe Betancourt, | Louisiana. |
| L. J. Blanchard, M. D., | Maine. |
| J. Henry Boger, | Pennsylvania. |
| Charles R. Brady, | Pennsylvania. |
| Rafael Chaguaceda, | Cuba. |
| Horace M. Christy, | Pennsylvania. |
| R. L. Culpeper, | West Indies. |
| L. F. Dayan, | New York. |
| Affonso H. DeMoura, | Brazil. |

John B. Dewees, ........................................................................Ohio.
B. K. Fetzer, ........................................................................Germany.
C. K. Fiske, ........................................................................Canada.
Emil Fuerth, ........................................................................Pennsylvania.
Eugene Goebell, ........................................................................Germany.
C. H. Goodrich, ........................................................................Minnesota.
Samuel A. Graham, Jr., ........................................................................Maryland.
John F. Hain, ........................................................................Pennsylvania.
H. R. Harbison, ........................................................................Pennsylvania.
C. J, Hazard, ........................................................................New Jersey.
Thomas K. Heaton, ........................................................................Pennsylvania.
C. T. Hewes, ........................................................................Illinois.
J. Calder Hinkle ........................................................................Pennsylvania.
G. W. Hosterman ........................................................................Pennsylvania.
T. L. James, ........................................................................Iowa.
R. E. Johnson, ........................................................................Ohio.
W. S. King, ........................................................................Tennessee.
Clara Kunast, ........................................................................Germany.
J. E. Libby, ........................................................................Pennsylvania.
John H. Lloyd, ........................................................................Pennsylvania.
H. S. Lowry, ........................................................................Ohio.
F. S. Maxwell, ........................................................................Ohio.
C. H. McCowan, ........................................................................Pennsylvania.
Louis A. Melze, ........................................................................Michigan.
F. M. Miller, ........................................................................Kentucky.
Walter H. Neall, ........................................................................Pennsylvania.
F. R. Newcomb, ........................................................................Pennsylvania.
C. E. Paddack, ........................................................................Ohio.
Jacob Perkins, ........................................................................Pennsylvania.
W. A. Phreaner, ........................................................................Pennsylvania.
W. M. Risdon, ........................................................................New Jersey.
Jas. W. Scott, ........................................................................Pennsylvania.
W. K. Sheafer, ........................................................................Pennsylvania.
William G. Smith, ........................................................................Maryland.
J. A. Suarez, ........................................................................Cuba.
Robert F. Swain, ........................................................................Pennsylvania.
D. S. Thomas, ........................................................................Pennsylvania.
J. L. Tierney, ........................................................................Pennsylvania.
W. E. Van Orsdel ........................................................................Pennsylvania.
H. L. Whitbeck ........................................................................New York.
F. D. Winchester ........................................................................Illinois.
E. N. Woodward ........................................................................California.
Harry Zimmerman ........................................................................Pennsylvania.

### UNIVERSITY OF TENNESSEE—DENTAL DEPARTMENT.

The annual commencement of this Institution was held February 24th, 1880.

The degree of D. D. S. was conferred on the following persons:

| NAME. | RESIDENCE. |
|---|---|
| Jas. C. Anderson, Jr | Tennessee. |
| Corydon W. Munson | Tennessee. |
| Benjamin Garrett | North Carolina. |
| George A McPeters | Tennessee. |
| James W. Williams | Georgia. |
| Byron B. Roberts | Tennessee. |
| William L. Smith | Georgia. |
| E. S. Chisholm | Alabama. |
| George T. Parker | Kentucky. |
| W. H. P. Jones | Tennessee. |
| A. F. Mallett | North Carolina. |
| J. J. Moore | Tennessee. |
| Joseph L. McGee | Tennessee. |

### PHILADELPHIA DENTAL COLLEGE.

The seventeenth annual commencement of the Philadelphia Dental College was held February 27th, 1880.

The degree of D. D. S. was conferred upon the following graduates, viz.:

| NAME. | RESIDENCE. |
|---|---|
| Napoleon B. Avery | Oregon. |
| William J. Bowden | Ireland. |
| Jacob S. Brandt | Pennsylvania. |
| Winslow L. Church | Rhode Island. |
| Edward G. Clark | Oregon. |
| William E. Davie | New Jersey. |
| Fred. I. Drowne | Massachusetts. |
| Ferdinand Egger | Germany. |
| James A. Finney | Pennsylvania. |
| Clarence E. Gates | Massachusetts. |
| S. Eldred Gilbert | Pennsylvania. |
| Oscar J. Gross | New Hampshire. |
| Alvin C. Harding | Nova Scotia. |

| | |
|---|---|
| John A. Hartman | Pennsylvania. |
| John V. Hemstreet | New York. |
| Reuben Herrod | Pennsylvania. |
| Alfred Hosfeld | Pennsylvania. |
| Harvey Iredell | New Jersey. |
| Albert H. Lewis | Pennsylvania. |
| William H. Marshall | Mississippi. |
| Milton C. Marshall | Mississippi. |
| J. Clyde Macartney | Pennsylvania. |
| Charles W. McConnell | Rhode Island. |
| W. Addison McKelvy | Pennsylvania. |
| Morton R. Metcalf | Minnesota. |
| Samuel A. Milton | Missouri. |
| Charles A. Page | Pennsylvania. |
| Walter Peake | England. |
| Fred. M. Smith | New York. |
| S. Goode Thomson | South Carolina. |
| Edwin R. Varcoe | New York. |
| William R. Webb | Pennsylvania. |

### NEW YORK COLLEGE OF DENTISTRY.

The fourteenth annual commencement of this Institution was held February 24th, 1880.

The degree of Docter of Dental Surgery was conferred on the following persons:

| NAME. | RESIDENCE. |
|---|---|
| Bernabe Arteaga | Cuba. |
| Virgilio Barranco | Cuba. |
| Hans F. L. Brauer | Germany. |
| Albert Jewett Butler | New York. |
| Emilio Dolores Costales | Cuba. |
| Wm. Curtis Deane | New York. |
| Wm. F. Davenport | New York. |
| Ewald O. H. Elsner | Germany. |
| Alfred Wells Edwards | France. |
| Wm. French Gerrish | Rhode Island. |
| Karl Grosch | Germany. |
| Karl A. W. Herrmann | Germany. |
| Frank Latson | New York. |
| Wm. Erik Lindstedt | New Jersey. |
| Herman Lambert | Poland. |
| Bryant Chas. Magennes | New Jersey. |

E. W. Knickerbocker ........................................................New York.
M. L. Obrieght........................................................New York.
August L. Peters........................................................New York.
George Willis Price........................................................California.
Sherman B. Price........................................................Connecticut.
Antonio B Primo........................................................Brazil.
David K. Reinhold........................................................New York.
Zachary T. Sailer........................................................New Jersey.
Alfred Russell Starr........................................................New York.
Wm. Elston Stelle........................................................Pennsylvania.
Oscar Schneider........................................................Germany.
George A. Walkley........................................................Massachusetts.

### DENTAL DEPARTMENT OF VANDERBILT UNIVERSITY.

The first annual commencement of this College was held February 23rd, 1880.

The degree of D. D. S. was conferred on the following persons, viz.:

| NAME. | RESIDENCE. |
| --- | --- |
| A. T. Kline | Tennessee. |
| D. L. B. Blakemore | Tennessee. |
| T. E. Cabaniss | Tennessee. |
| R. B. Lees, M. D. | Tennessee. |
| J. H. Webber | Tennessee. |

### DENTAL DEPARTMENT OF THE UNIVERSITY OF MICHIGAN.

The fifth annual commencement of this department was held March 24th, 1880.

The degree of D. D. S. was conferred upon the following persons, viz.:

| NAME. | RESIDENCE. |
| --- | --- |
| William Berry Armendt, | Kentucky. |
| Thomas Wesley Beckwith, | Illinois. |
| Uriah Dildine Billmeyer, | New York. |
| William Fairman Bradner, | Michigan. |
| George Heart Brown, | Michigan. |
| David Ethelbert Callaghan, | Japan. |
| John Peter Carmichael, | Illinois. |
| Suel Erastus Clark, | New York. |

Williams Donnally,........................................................Kentucky.
Hiram Edgar Dunn,........................................................Pennsylvania.
Alma W. E Fuellgraff,........................................................Germany.
Frank F. Hoyer,........................................................New York.
Alfred Wright Hoyt,........................................................Wisconsin.
Ormand Courtland Jenkins,........................................................Michigan.
Aaron Churchill Johnson,........................................................Michigan.
George Frederick Kimball,........................................................Michigan.
Thomas Chalmers Leiter,........................................................Ohio.
Amos Marion Long,........................................................Michigan.
Frank Flavius Little,........................................................Ohio.
Ossian C. Moon,........................................................Indiana.
Arthur Clayton Nichols,........................................................Michigan.
Evelyn Pierrepont,........................................................England.
Douglas Potterf,........................................................Ohio.
William John Poyser,........................................................Ohio.
John Nelson Reynolds,........................................................Pennsylvania.
Collins McKnight Roe,........................................................Ohio.
Maurice James Sullivan,........................................................California.
Ira Emmit Sampsell,........................................................Ohio.
Charles Edwin Stroud,........................................................Ohio.
Immer Crittenden St. John,........................................................Minnesota.
Oliver Stacy Voak,........................................................New York.
Julius Charles Waldron,........................................................Ohio.
Wilbur Silvius Whisler,........................................................Ohio.
Robert Addison Young,........................................................Michigan.

### INDIANA DENTAL COLLEGE.

The first annual commencement of the Indiana Dental College was held March 10th, 1880.

The degree of D. D. S. was conferred on the following graduates:

| NAME | RESIDENCE. |
|---|---|
| R. W. Van Valzah | Terre Haute, Indiana. |
| W. E. Swigert | New London, Missouri. |
| E. J. Church | Laporte, Indiana. |

